# Apoptosis of proximal stump postganglionic brachial plexus injury, before and after six months post-trauma

**DOI:** 10.1016/j.amsu.2021.02.002

**Published:** 2021-02-17

**Authors:** Gana Adyaksa, Heri Suroto

**Affiliations:** Department of Orthopedics & Traumatology, Faculty of Medicine, Universitas Airlangga / Dr. Soetomo General Academic Hospital, Surabaya, Indonesia

**Keywords:** Postganglionic brachial plexus injury, Apoptosis, Proximal stump

## Abstract

**Background:**

The success of the micro-surgery procedure for the treatment of postganglionic brachial plexus injury was influenced by several factors, including the surgical timing and also the viability of the proximal stump. This study evaluates the evidence of apoptosis in the brachial plexus proximal stump and its correlation with the surgical timing.

**Methods:**

Proximal stump biopsy of postganglionic brachial plexus injury patients were obtained during nerve procedure surgery. The samples were grouped based on the surgical timing, before six months post-trauma (early group) and after six months post-trauma (late group). The apoptosis of motorneurons was evaluated by immunohistochemistry expression of Caspase-3, TNF-α, Caspase-8, and Caspase-9.

**Results:**

Immunohistochemistry findings showed higher expression of Caspase-3 in the late group compared to the early group, as well as the expression of Caspase-8 and Caspase-9 (p < 0,05), and with a positive correlation between Caspase-8 and Caspase-9 to Caspase-3. Meanwhile, TNF-α expression was higher in the early group than the late group (p < 0,05) and with no correlation between TNF-α to Caspase-3.

**Conclusion:**

Apoptosis of proximal stump motorneuron plexus brachialis on more than six months post-trauma is higher than on less than six months post-trauma.

## Highlights

• Study of apoptosis of the brachial plexus proximal stump of the postganglionic BPI.
• Compare between before 6 months post-trauma and after 6 months post-trauma.
• Evaluate the apoptosis, the extrinsic and intrinsic pro-apoptosis pathway.
• The apoptosis of more than 6 months post-trauma is higher than before 6 months.

## 1 Introduction

Brachial plexus injury (BPI) is a severe trauma that generally occurs in young and productive age patients, usually due to a traffic accident and an injury to the area between the neck and shoulders [1,2]. This injury causes loss of function and ability to carry out their daily activities and work activities, which will impact job loss, economic decline, depression, anxiety, and even suicide [3,4].

There are currently several surgical options for BPI, including nerve graft, nerve transfer, and functional muscle transfer [[5], [6], [7]]. The success of this micro-surgery procedure is influenced by several factors, including the timing of surgery. A systematic review study showed that BPI's surgical procedure performed less than six months after the trauma has a better outcome than if performed after six months post-trauma [8]. This is usually caused by motor endplate degeneration of more than six months of denervated muscle [9,10]. Furthermore, there was evidence that after six months post-trauma, the nerve regeneration capacity was significantly reduced [11].

In postganglionic brachial plexus injury, nerve grafting is one alternative surgical procedure to restore some muscle functions [12]. In nerve grafting, the proximal stump's viability is essential to ensure the success of the grafting. After trauma, the brachial plexus proximal stump could undergo some particular event, depends on the involved damaged area. The breakdown of the proximal stump is limited and typically only progresses to the first node of Ranvier. If the site of injury is close to the neuronal body, apoptosis may occur [13].

Apoptosis of neural cells could be triggered by two major principal pathways: the intrinsic (or mitochondrial) pathway and the extrinsic (or death receptor) pathway. The extrinsic apoptosis pathway is triggered by the ligation of tumor necrosis factor (TNF)-family death receptors at the cell surface, then recruit an adapter protein, TNF-R-associated death domain (TRADD), and activate the pro-apoptosis caspase-8. The intrinsic (mitochondrial) pathway of apoptosis is triggered within the cell, causing expression or activation of BH3-only proteins that activate Bax to form pores in the outer mitochondrial membrane, releasing cytochrome c to bind APAF-1, activating caspase-9. Finally, both caspase-8 or caspase-9 activate downstream of caspase-3 as the apoptosis executor [[14], [15], [16]].

To date, there was no study evaluating the apoptosis of the proximal stump motorneuron in postganglionic brachial plexus injury and its relation to the surgical timing STS. This study aims to evaluate the evidence of apoptosis in the brachial plexus proximal stump and its correlation to surgical timing in postganglionic BPI patients and further investigate the possible pathway of the occurred apoptosis.

## 2 Methods

### 2.1 Type of study and sample

This research is a cross-sectional study. Proximal stump of brachial plexus biopsy from 20 postganglionic brachial plexus injury patients was obtained during brachial plexus nerve surgical procedures. The inclusion criteria for the samples are >18 years old postganglionic BPI patients that underwent nerve surgical procedures (nerve transfer, nerve grafting, or free functional muscle transfer) and excluded if there is a history of infection. They grouped based on the surgical timing, comprised of 11 patients in the early group (before six months post-trauma) and nine patients in the late group (after six months post-trauma). The written informed consent for biopsy was obtained from the patients. This study already had ethical clearance from the Health Study Ethical Committee of Dr.Soetomo General Hospital Surabaya, East Java, Indonesia.

### 2.2 Immunohistochemistry staining

The biopsy tissue is then fixed with 10% formalin and embedded in paraffin for immunohistochemistry staining. Immunohistochemistry staining was performed using Active Caspase-3 Monoclonal Antibody (Bioenzy), TNF-α Monoclonal Antibody (Bioenzy), Caspase-8 Polyclonal Antibody (Bioenzy), and Caspase-9 Polyclonal Antibody (Bioenzy) according to manufacture protocol.

The study was presented in line with the STROCSS criteria [17] and already registered in the Chinese Clinical Trial Registry, and the unique identifying number (UIN) is ChiCTR2000039719 [18].

### 2.3 Data collection and analysis

A section from each sample's nerve stump in each group was photographed using a digital camera and tested microscopically. Ten randomly selected regions from each slice were evaluated for Caspase-3, TNF-α, Caspase-8, and Caspase-9. Under 400x magnification of a microscope, the motorneuron cell with brown cytoplasm was counted as Caspase-3/TNF-α/Caspase-8/Caspase-9 positive cell for each staining.

### 2.4 Statistical analysis

The mean and standard deviation of Caspase-3/TNF-α/Caspase-8/Caspase-9 positive cell of each group was calculated. Comparison analysis by independent *t*-test and correlation analysis by Pearson were performed using SPSS version 21. The difference in the expression of each parameter was regarded as statistically significant at p-value <0,05.

## 3 Results

There were 11 patients in the early group and nine patients in the late group, with the average time to surgery was 3,18 months and 32 months, respectively. In both groups, most of the biopsy samples were obtained from the C5 root of the brachial plexus. Majority of the patients was male, and all of the injuries were caused by traffic accident (Table 1).

**Table 1:** Sample's characteristics.

	Early Group	Late Group
**Sample number**	11	9
**Gender**	Male = 10	Male = 8
	Female = 1	Female = 1
**The average age**	25 yo	29.9 yo
**Mode of Injury**	Motor Vehicle Accident (100%)	Motor Vehicle Accident (100%)
**The average time to surgery (SD, Min-Max)**	3.18 months (3,18; 1–6)	32 months (14,52; 10–62)
**Biopsy location**	C5 = 8, C6 = 3	C5 = 7, C6 = 1, C7 = 1

**Fig. 1:**
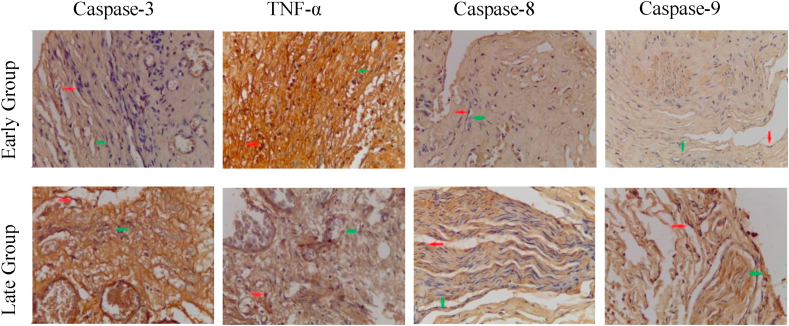
Immunohistochemistry staining of Caspase-3, TNF-α, Caspase-8, and Caspase-9 expression on brachial plexus proximal stump, in the early group and late group. 400x magnification. (Red arrow = IHC positive cell. Green arrow = IHC negative cell). (For interpretation of the references to colour in this figure legend, the reader is referred to the Web version of this article.)

**Fig. 2:**
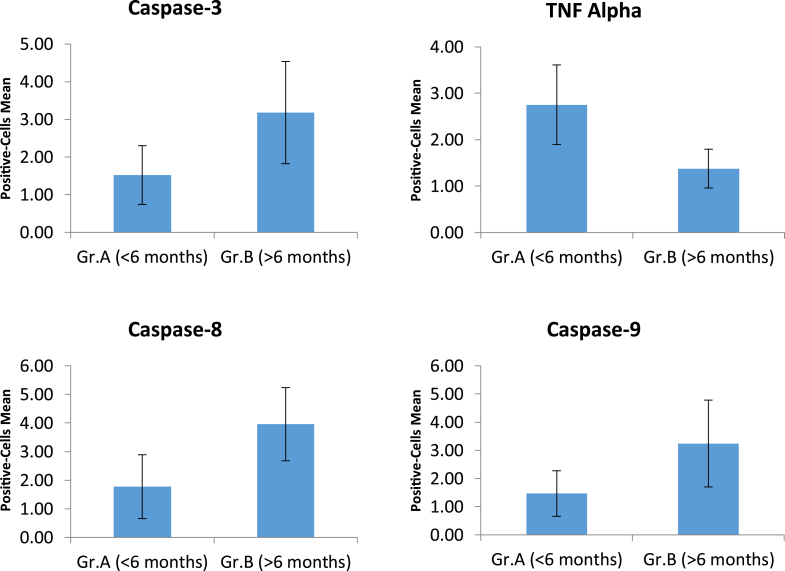
The immunohistochemistry expression analysis of Caspase-3, TNF-α, Caspase-8, and Caspase-9.

The average of Caspase-3 positive cells, with brown cytoplasm (Fig. 1), on the early group (1.52 ± 0.78), was lower compared to on the late group (3.18 ± 1.36) and statistically significant (p = 0.03) (Table 2 and Fig. 2). This suggesting that motorneuron apoptosis was higher in the late group (after six months post-trauma).

**Table 2:** The immunohistochemistry expression and comparison analysis of Caspase-3, TNF-α, Caspase-8, and Caspase-9.

Variable	Group	N	Mean ± SD	p^a^
**Caspase-3**	Early (<6 months)	11	1.52 ± 0.78	p = 0.003
	Late (>6 months)	9	3.18 ± 1.36	
**TNF-α**	Early (<6 months)	11	2.75 ± 0.86	p = 0.001
	Late (>6 months)	9	1.38 ± 0.42	
**Caspase-8**	Early (<6 months)	11	1.77 ± 1.12	p = 0.001
	Late (>6 months)	9	3.96 ± 1.28	
**Caspase-9**	Early (<6 months)	11	1.47 ± 0.81	p = 0.004
	Late (>6 months)	9	3.24 ± 1.54	

a Independent T-Test.

The average of TNF-α positive cells in the early group (2.75 ± 0.86) was higher than in the late group (1.38 ± 0.42) and statistically significant (p = 0.03) (Table 2 and Fig. 2). This suggests that the expression of TNF-α as an inflammatory mediator and the extrinsic apoptotic pathway's initiator was higher in the early phase of trauma (less than six months post-trauma) than in the late phase.

**Fig. 3:**
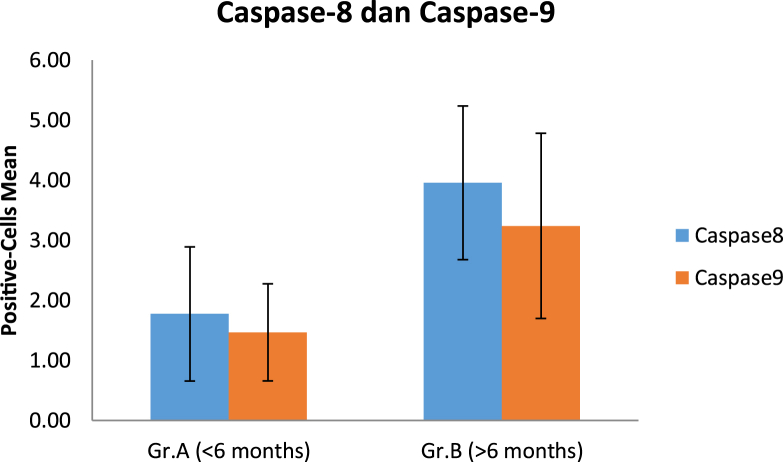
The comparison of the Caspase-8 and Caspase-9 expression on early and late group.

The Caspase-8 and Caspase-9 positive cells in the early group (1.77 ± 1.12 and 1.47 ± 0.81, respectively) were lower than in the late group (3.96 ± 1.28 and 3.24 ± 1.54) and statistically significant (p = 0.001 and p = 0.004) (Table 2 and Fig. 2). For the comparison between Caspase-8 and Caspase-9, the expression of Caspase-8 was higher than Caspase-9, both in the early and late group, but without statistically significant difference (p > 0.005) (Table 3 and Fig. 3).

**Table 3:** Comparison of the Caspase-8 and Caspase-9 expression on early and late group.

Group	Variable	N	Mean ± SD	p^a^
**< 6 months**	Caspase-8	11	1.77 ± 1.12	p = 0.212
	Caspase-9	11	1.47 ± 0.81	
**> 6 months**	Caspase-8	9	3.96 ± 1.28	p = 0.217
	Caspase-9	9	3.24 ± 1.54	

a Independent T-Test.

Furthermore, in correlation analysis, there was no correlation between Caspase-3 expression and TNF Alpha expression. The number of Caspase-3, Caspase-8, and Caspase-9 positive cells was higher in the late group, and in the correlation analysis, there was a positive correlation between Caspase-8 and Caspase-3, and also between Caspase-9 and Caspase-3. (Table 4).

**Table 4:** Correlation analysis of the TNF-α, Caspase-8, and Caspase-9 to the Caspase-3 expression.

Variable	N	Correlation test (Pearson)
**TNF-α**	20	p = 0.075; r = - 0.407
**Caspase-3**	20	
**Caspase-8**	20	p = 0.000; r = 0.792
**Caspase-3**	20	
**Caspase-9**	20	p = 0.008; r = 0.575
**Caspase-3**	20	

## 4 Discussion

In the BPI's microsurgical procedure, one of the essential factors determining the nerve procedure's success is the survival of the involved nerve's proximal stump. To date, the apoptosis of the proximal stump of the BPI patients and its relation with the surgical timing is still unclear.

The result of this study showed that apoptosis of motorneuron was undergone in the proximal stump of the brachial plexus, proven by the finding of the Caspase-3 positive cell as a marker of apoptosis event. Furthermore, the motorneuron's apoptosis level was higher in patients who had trauma for more than six months. It suggests that the apoptosis event in the brachial plexus is a timely fashion, which can become a consideration factor for determining the optimal time for nerve-related surgery.

TNF-α is one of the inflammatory mediators playing a role in the systemic response to trauma and infection. It regulates inflammatory responses after the injury to the peripheral or central nervous system and initiates the activation cascade of other cytokines and growth factors [18]. This might cause the expression of TNF-α in the <6 months the post-trauma group was higher than in the >6 months post-trauma group.

In apoptosis, the TNF-α′s biologic activity depends on the activity of their different membrane receptors, namely TNFR1 and TNFR2, which both have different signaling but also overlap [18]. The binding of TNF Alpha to the TNR1 can activate the survival pathway of NF-κB and even the caspase-dependent cell death. The binding of TNF Alpha to the TNFR1 activates the TNFR1, then binds the TRADD (TNFR-associated death domain) and bind the TRAFs (TNFR-associated factor) and RIP to activate the NF-κB (Complex I), which is a survival pathway or anti-apoptosis. In the second step, TRADD and the kinase RIP1 associated with FADD (Fas-associated protein with death domain) and Caspase-8 form a cytoplasmic complex (Complex II), resulting in cell death [20,21].

This study found that the expression of TNF-α was not correlated with the expression of the Caspase-3 as an apoptosis marker. Furthermore, even though TNF alpha expression was higher in the early group than in the late group, the expression of pro-apoptosis caspase-8 and the apoptosis marker caspase-3 precisely low. This is following the result of the Harper et al. study, which states that Caspase-8 and FADD were not recruited to a TNF-induced membrane-bound receptor signaling complex as occurs during CD95 (Fas) or TRAIL (TNF-related apoptosis-inducing ligand) signaling, but instead must be activated elsewhere within the cells [22].

There was no significant difference in expression between Caspase-8 and Caspase-9, both in the early and late groups, so we cannot conclude which one of the apoptosis pathways that are more dominant in initiating apoptosis cascade on brachial plexus injury. Although initially suggested that the death receptor apoptosis pathway initiated through Caspase-8 and the mitochondria-mediated apoptosis pathway through Caspase-9 were two independent pathways, there was cross talk interaction between them. In particular conditions, Caspase-8 could cleave the cytosolic bid (BH3-interaction death domain agonist, one of the Bcl-2 family) and translocate to mitochondria and mediates the release of cytochrome c, and activates the Caspase-9 [21,23].

This limitation of this study is the limited sample number and limited surgical time grouping. Furthermore, more apoptotic proteins pathway, such as FAS, TNF-related pathway, and mitochondrial pathway, also needs to be explored to get a more comprehensive understanding of apoptosis in BPI.

## 5 Conclusion

Apoptosis of proximal stump motorneuron plexus brachialis on more than six months post-trauma is higher than on less than six months post-trauma. This further supports the importance of performing nerve-procedure surgery in postganglionic BPI patients as early as possible to obtain the optimal result.

## Funding/support statement

This research did not receive any specific grant from funding agencies in the public, commercial, or not-for-profit sectors.

## Provenance and peer review

Not commissioned, externally peer-reviewed.

## Declaration of competing interest

The authors declare no conflict of interest in this study.
